# The intervention design to promote active travel mode among children and adolescents: A systematic review

**DOI:** 10.1016/j.heliyon.2024.e26072

**Published:** 2024-02-13

**Authors:** Kattreeya Chanpariyavatevong, Thanapong Champahom, Chamroeun Se, Sajjakaj Jomnonkwao, Vatanavongs Ratanavaraha

**Affiliations:** a Department of Transportation Engineering, Suranaree University of Technology, 111, University Avenue, Suranari, Muang, Nakhon Ratchasima, 30000, Thailand; b Department of Management, Faculty of Business Administration, Rajamangala University of Technology Isan, 744, Sura Nari Rd, Nai-muang, Muang, Nakhon Ratchasima, 30000, Thailand

**Keywords:** Active travel mode, Adolescents, Children, Intervention, School travel, Systematic review

## Abstract

**Background:**

Walking and cycling are examples of active travel modes or nonmotorized modes that rely on human physical power rather than fossil fuel consumption. In the context of short-distance journeys, active travel modes are advocated as feeder modes to reduce energy consumption and CO_2_ emissions. However, in Thailand and many other developing countries, these modes of transportation have not been widely adopted or effectively promoted. The absence of comprehensive campaigns and interventions to promote and facilitate the use of active travel modes has become a major barrier to their adoption, particularly among adolescents who will be future global citizens. Therefore, a campaign or intervention targeting adolescents is imperative to introduce and persuade them to adopt active travel modes. This study aims to provide guidelines for developing a robust intervention strategy to promote active travel modes among adolescents.

**Methods:**

This study performed a systematic review to achieve the research goal using a particular search and selection approach. The search strategy has focused on published studies in the English language since 2014 to highlight the most recent trends. The selection process focused on articles relevant to promoting active travel mode among children and adolescents (up to 18 years old) through intervention.

**Conclusions:**

A total of 16 studies were included. The findings reveal that successful interventions to promote active travel modes consist of an educational program and activities incorporating gamification to encourage their use. Furthermore, the intervention should last longer than one month to be effective.

## 1 Introduction

In recent years, people have become interested in their physical health. To be healthy, nourishment and exercise habits are important [1]. Several studies have confirmed that exercise or physical activities can reduce mortality risk [2,3]. Correspondingly, World Health Organization [4] revealed that physical inactivity is the fourth leading risk factor for global mortality, people who walk or cycle a minimum of 150 min a week can reduce the risk of mortality by 10%. Walking and cycling were defined as active travel modes [[5], [6], [7], [8], [9]], which depended on human physical power [10]. Furthermore, active travel mode plays an important role in reducing the risk of cardiovascular disease and other chronic diseases [[11], [12], [13]].

In many developed countries, the active travel mode is considered the simplest way to travel for daily short-distance trips, not exceeding 1.5 km [14]. Using active travel mode for short-distance journeys is the easiest and most practical way to reach the destination or connect to other long-distance modes (feeder mode). In areas with traffic congestion, active travel mode might allow travelers to move faster than using motorized vehicles for short-distance journeys, thereby helping them save travel time. Using active travel modes instead of motorized travel modes reduces CO_2_ emissions from incomplete combustion, which is the main cause of air pollution and climate change [11,15,16]. The effects of climate change have been clearly evident in several areas and increase in severity each year [16]. In addition to the circumstances in Thailand, the overall level of CO_2_ emissions tends to increase yearly, especially in the transportation sector with approximately 80 million tons in 2022 [17]. Thailand has faced serious air pollution in the past three years. Switching from motorized to nonmotorized travel mode in short-distance journeys might be an empirical approach to mitigate this problem. Hence, promoting campaigns to reduce motorized vehicles is necessary. Shifting from a motorized to a nonmotorized travel mode for short-distance journeys reduces CO_2_ emissions and fuel consumption and provides various economic and health advantages to travelers. These advantages include reduced travel time, fuel cost savings, and the promotion of physical and mental well-being. Many developed countries have implemented campaigns to encourage active travel modes for short-distance journeys as a sustainable solution, aimed at alleviating air pollution and related issues sustainably [18]. This is because it is the most cost-effective mode of transportation with numerous associated benefits.w.

Many campaigns or interventions have promoted the active travel mode that have succeeded widely worldwide, such as the School Travel Plan (STP) in Canada [[19], [20], [21]]. During the intervention, many activities and mini-campaigns were offered, including educational programs (e.g., safe ways to bike and walk, bicycle and walk days) and activities that incorporated a game concept, thus significantly changing the attitudes and travel behavior [22]. Asian countries, such as Singapore, China, and the Philippines, have launched active travel campaigns to encourage people to travel, particularly young people, to walk and bike as a feeder mode, and to build a greater awareness of environmental sustainability [[23], [24], [25], [26], [27]]. However, there is no obvious evidence that the existing intervention successfully changed the travel behavior of the participants in an Asian country. Furthermore, many studies have confirmed that changing the adolescents’ attitudes and behaviors toward active travel mode is significantly more possible than adults [[28], [29], [30]]. Therefore, the intervention should focus on promoting active adolescent travel mode.

In Thailand, existing interventions to promote active travel modes have been designed for adults, who may be more resistant to changing their attitudes and behaviors [31]. Furthermore, the intervention or campaign appears ineffective and inappropriate, incapable of changing long-term travel behavior. Therefore, Thailand lacks the potential for an appealing intervention or campaign to promote active travel mode. The intervention developed in Europe was found to be difficult to adapt in different contexts due to variations in built environments and travel behavior. Furthermore, few studies have been related to the principles and design of appropriate interventions that focus on specific areas. To fill this research gap, the present study aims to investigate interventions, including concepts and principles, to promote an active travel mode suitable for Thailand's context. This study is intended to guide the design of appropriate interventions to promote active travel mode in different areas and built environments.

### 1.1 Theoretical fundamentals

Active travel mode refers to any form of transportation that involves physical activity, such as walking or cycling, rather than relying on motorized vehicles [32,33]. Active travel modes have multiple benefits, including improvements in health, environmental impact, and behavioral change. Regarding individual benefits, research has demonstrated a heightened interest in maintaining personal health and increasing physical activity levels. Regular physical activity can prevent illness and reduce the risk of cardiovascular diseases [12,34]. Additionally, shifting to nonmotorized travel modes as part of transportation can significantly reduce traffic congestion, a major source of CO_2_ emissions [[35], [36], [37]]. To contribute positively to society, people can use active travel modes for short-distance travel and help support cities reduce air pollution and maintain fresh air, which is essential to human well-being and creates a better environment for communities. Although active travel modes have become increasingly popular in developed countries [38] and offer numerous benefits to travelers, the environment, and communities, the fact remains that they are not as convenient as motorized travel modes [39]. Some travelers may not be willing to abandon the convenience of motorized travel and switch to active travel modes. Therefore, an effective campaign or intervention is needed to promote active travel modes.

Interventions are critical in encouraging active travel modes commonly used in the target population. The intervention principle may differ depending on the target group [30].Therefore, the educational program aims to provide fundamental knowledge about the benefits of active travel modes and examples of their positive and negative impacts. Furthermore, the fundamental competencies, such as walking and cycling in the city and basic knowledge of road safety, including understanding traffic regulations and signs, must be included to ensure the effectiveness of the educational program. This safety content in the intervention can help participants avoid accidents on the road during their travel [40]. Moreover, applying the knowledge gained from educational programs in real-world situations is crucial. The intervention should incorporate fun activities, such as games and challenges, to facilitate this process. These activities promote the replication of active travel behavior and boost traveler confidence in using active modes of transportation. Typically, the most basic activities or challenges provided as interventions are walking/cycling games, which an educator monitors. In addition, other activities, such as walking or cycling day challenges and walk rally games, encourage the adoption of active travel modes. When children and adolescents are familiarized with these activities, barriers to using active travel modes can be reduced. In many countries, interventions are integrated into educational curricula, such as the STP in Canada [19,41]. The STP initiative, run by a non-profit organization committed to nonmotorized travel, offers intervention programs to schools nationwide, which are monitored by specialists to ensure that participants gain the necessary knowledge and participate safely [42].

In terms of changing travel behavior, interventions can significantly impact the change of travel behavior. Interventions can help create an environment that encourages and supports active travel mode, thereby assisting people to shift away from motorized travel mode and even change their attitude toward active travel mode. These changes in travel behavior and attitude may include the following:

- *Increased frequency of active travel*: Interventions can encourage people to walk or cycle more frequently by making it easier and more attractive.
- *Longer travel distances*: Interventions can help people feel more comfortable and confident in traveling longer distances by active modes of transportation, such as by providing safe and efficient bike lanes, pedestrian paths, and other supported infrastructures.
- *Reduced car usage*: Interventions can help to reduce reliance on motorized vehicles, resulting in fewer car trips and less traffic congestion on the roads.
- *Increased use of public transportation*: By encouraging active travel, interventions can encourage people to use public transportation more frequently as part of their travel behavior.

In addition, the intervention can be informed by various behavior change theories, such as the health belief model and the theory of planned behavior [43,44]. These theories suggest that interventions should focus on increasing awareness, providing social support, and improving self-efficacy to promote behavior change.

In conclusion, an intervention does not follow a fixed pattern and varies based on the specific area and the residing people's natural characteristics. Effective interventions to promote active travel mode and change travel behavior can require government support, such as policy changes, encouragement of social support, and improvements in infrastructure [12]. However, evidence indicates that interventions can positively impact travelers' behavior by persuading them to change their travel behavior, including their attitudes toward active travel mode. This is due, in part, to the safety contexts provided by interventions, which can make travelers feel more comfortable and secure when using active travel mode [19,45,46].

## 2 Materials and methods

This study used the systematic review approach to investigate an effective intervention aimed at promoting active travel mode among adolescents. The Preferred Reporting Items for Systematic Review and Meta-Analyses (PRISMA) checklist was used for the systematic review, as shown in the S1 file. The following section will discuss the search strategy and selection process.

### 2.1 Search strategy

In this study, the literature search was conducted using three electronic databases: Scopus, Wiley online library, and ScienceDirect. Since the keywords or specific terms used for the search are a crucial factor in providing effective results, the keywords were collected and suggested by the researchers’ expertise. Four keywords (i.e., active travel mode, adolescents, intervention, and school travel) were used for searching to meet the target studies. Searching using these keywords yields particularly fascinating studies. Since 2014, the number of research articles relevant to interventions in active travel modes among adolescents has increased rapidly and is expected to continue to increase, demonstrating a growing trend in this type of research. This is in line with the worldwide interest in sustainable mobility. Therefore, all English-language publications from 2014 to the present were included, whereas prior publications and information from before 2014 were excluded to update the latest trends. Any studies excluded from these criteria were discussed among the authors, generally unselected.

### 2.2 Selection and review process

A certain number of studies or research articles are relevant to the active travel mode and intervention, but not all these studies were selected. The selection criteria are as follows:

***i.*** emphasized for children and adolescents under 18;
***ii*.** contained an intervention to promote the active travel mode, including travel to school or a daily short trip; and
***iii*.** provided the results of the mentioned interventions for promoting active travel mode.

These criteria were established to initially exclude irrelevant research articles and obtain the most pertinent ones. Studies that did not meet these criteria were not selected. Furthermore, each selected study was required to contain descriptive data on the sample or participants, an empirical objective, details on the intervention strategies, and the results.

## 3 Selection procedure

The first author comprehensively searched for articles or records in the specified database, which were subsequently imported into EndNote X9. During this process, duplicate records were carefully removed. Unduplicated articles were independently screened (title and abstract) and assessed in full text for eligibility by the first two authors. Moreover, to ensure rigorous evaluation and agreement, the first two authors synthesized and discussed the selected articles. Their goal was to reach an agreement on the inclusion of relevant studies. If a consensus could not be reached, the third and fourth authors were involved to further deliberate on the full-text articles and reach a final decision.

## 4 Results

**Fig. 1:**
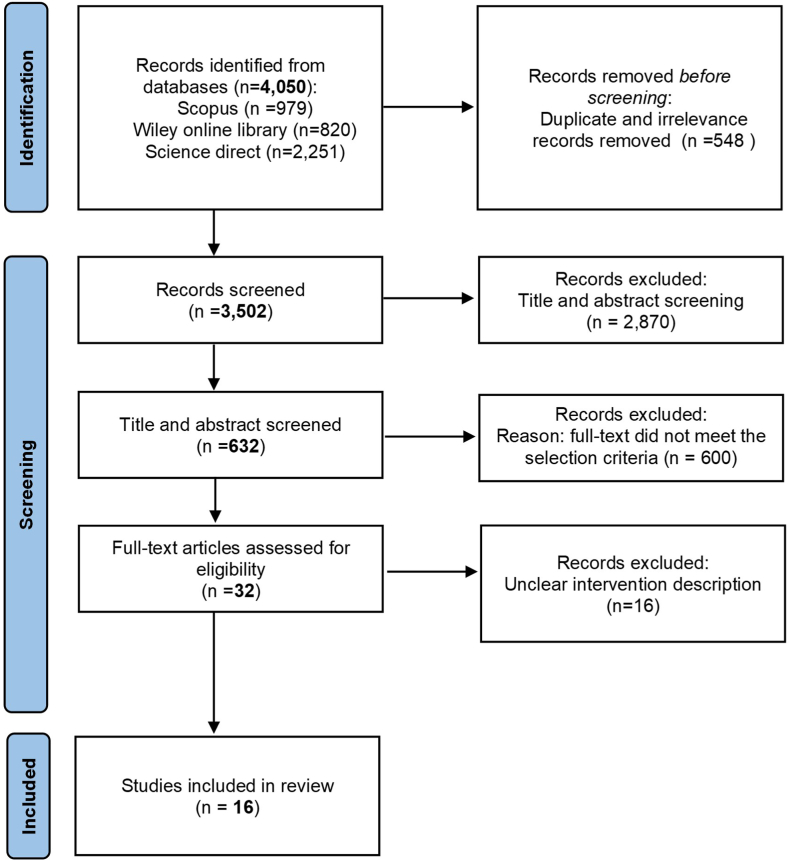
The PRISMA flow chart for literature search and selection process.

According to the previous part's search strategies from electronic databases, 4050 articles were found: 979 from Scopus, 820 from Wiley online library, and the remaining 2251 from ScienceDirect. There were 548 duplicated articles, leaving 3502 articles. A selection process was carried out after obtaining many relevant articles during the searching process. A total of 632 articles with screened titles and abstracts were chosen, and articles with titles and abstracts unrelated to active travel mode interventions were excluded. Subsequently, a full-text assessment was conducted, and articles that did not meet the selection criteria were removed, resulting in a final set of 32 articles. Nevertheless, the detailed intervention part was supposed to be clearly indicated, and 16 articles were excluded due to unclear intervention description. Ultimately, 16 articles were included in this study. The literature search and selection process flow diagram is shown in Fig. 1. The 16 articles related to the intervention to promote active travel are presented in Table 1. These studies were carried out in Europe, Americas and Australia with variety of countries, Austria, Germany, Ireland, Sweden, Denmark, Scotland, Belgium, Canada, the United States, the United Kingdom, and Australia. Most studies were conducted in Europe, with 10 studies. All studies focused on implementing the intervention in children or adolescents under 18 years of age who were in elementary, primary, and secondary schools.

**Table 1:** Findings of the reviewed sources (N = 16).

Authors	Origin	Purpose	Target population	Implementation period	Intervention strategies	Outcomes
Bungum, Clark [45]	USA	Examine the impact of a one-day intervention on the changeability of children's travel behavior.	Primary school students	1 day	- Educational program: pros and cons of active travel mode - Activities: motivate to use active travel mode as school travel mode	The one-day intervention was notable for changing the travel behavior of the intervention group sample, increasing active travel to and from school by approximately 10.3%. However, the effect of the one-day intervention was temporary; a week later, the active travel rate returned to the baseline level.
Christiansen, Toftager [47]	Denmark	To validate the influence of physical activity intervention on adolescents' active travel behavior.	Secondary school students	2 years	- Performing an after-school fitness program. - Conducting a campaign to encourage parents to minimize school trips by motorized vehicles. - Improving the infrastructure around the school to support active transport to the school.	The estimated portion of active travel mode in adolescents at follow-up after intervention increased slightly from the baseline level.
Ducheyne, De Bourdeaudhuij [48]	Belgium	To evaluate the impact of short-term cycling skill training on children.	Secondary school students.	5 months	- Cycling training course (i.e., walking with bicycle, cycling on the straight line, and cycling between obstacles) - The concepts of games were integrated into the training course for fun reasons.	After five months of follow-up, the children's cycling skills across the intervention groups were significantly improved.
Mammen, Stone [20]	Canada	To evaluate the intervention in Canadian schools, examine the average change in the mode of the school travel.	Primary school students	1 year	- The School Travel Plan (STP) was provided as an intervention. - Educational programs and workshops - Walk-to-school group activities and other enforcement strategies were included.	The intervention effectively changed the mode of travel in Canadian schools to active travel, with a large rate of approximately 80%.
McDonald, Steiner [55]	USA	To distinguish the impacts of Safe Routes to School (SRTS) programs on increasing walking and bicycling among children.	Secondary school students	5 years	- SRTS programs were conducted as an intervention. - Education programs: classroom safety instruction and skills workshops outside of the classroom. - Activities: games and challenges, small rewards were offered to encourage students to walk or cycle to school buses. - Improving facilities related to walking and cycling for safety.	The results indicated that more than 31% of the students switched to walking and cycling to school after five years of participating in SRTS programs.
McMinn, Rowe [46]	Scotland	To investigate the impact of the intervention (Traveling Green program) on Scottish children.	Primary school students	6 weeks	- Traveling Green was promoted as an intervention - Teacher's handbook with 13 lessons (e.g., meaning, benefits of active transport including road safety). - The children's pack contains guidebook description the campaign and booklet for recording travel behavior.	In follow-up, the intervention (Traveling Green) was significantly increased active travel among samples. Most of the samples tend to increase their walking or cycling, reducing the use of cars.
Denstel, Broyles [56]	Worldwide	To determine whether Active School Transport (AST) increases the time that students spend on physical activity.	Primary school students	3 months	- AST was implemented as an intervention in this study. - The body mass index and travel diary were collected to compare before and after the program.	The intervention significantly increased the level of physical activity among participants, demonstrating a positive behavioral change.
Coombes and Jones [57]	UK	To evaluate the impact of interventions to change children's physical activity levels.	Primary school students	9 weeks	- The Beat the Street program was implemented as an intervention. - Participants were instructed to touch the provided “Beat box” when walking or cycling to obtain points. - Weekly prizes were provided to participants with the most points each week.	Beat the Street program implemented in the samples can increase the frequency of active travel commuter to school in post-intervention compared to pre-intervention.
Murtagh, Dempster [49]	Ireland	To identify the maintenance of the intervention that promotes AST.	Primary school students	4 years	- AST was provided as an intervention. - Walking/cycling campaign - Competition relevant to active transport to school.	Children who live in urban areas are likely to maintain active travel to school. The increasing distance from residence to school harms children's maintaining active travel behavior.
Verhoeven, Simons [54]	Belgium	Examine the impact of the intervention on the intention to use active travel mode among adolescents.	Secondary school students	1 week	- One-week intervention. - Active Transport Lesson and Practicalities Planning of Active Transport	Unfortunately, the intervention did not increase the intention to use active travel mode. Intervention strategies may not motivate enough.
Villa-González, Ruiz [52]	USA	Identify the effects of intervention on active behavior in children.	Primary school students	6 months	- Educational program: perception of safety on the way to school. - Activities: telling stories, performing mini shows related to active travel mode, and exploring by walking around the school.	In the follow-up step, it was illustrated that the active transport of the experimental group to school increased markedly even beyond the intervention period.
Lambe, Murphy [53]	Ireland	To extend the impacts of promoting active transport intervention on primary students.	Primary school students	2 years	- Educational programs: introduction to safer walking routes to school - Activities: bike/walking day, walking and taking the bus to school, and holding the seminar to encourage active travel to school.	Overall there was no outstanding change in active travel behavior in children in both two intervention towns, no difference between the intervention towns and the control town.
Stark, Berger [30]	Austria and Germany	Examine the effects of an intervention on children's changes in travel attitude intentions and behavior.	Secondary school students	1 year	- The researchers designed the intervention. - Using three principles; information (educate and transfer knowledge), reflection (record travel diary school - home) and actions (games, challenge, walk day and bicycle day)	The provided interventions and activities were significantly effective in changing attitude and behavior toward active travel of the test group, intending to use nonmotorized mode more and motorized less.
Buttazzoni, Clark [19]	Canada	Examine the impacts of the intervention that promotes active travel to school toward children's travel behavior, including parents' perception of active travel barriers.	Primary school students and their parents	5 months	- STP was provided to the samples as an intervention. - Educational program: how to plan a safe walking/cycling route. - Activities: games related to active travel, active day campaign.	The intervention successfully changed parents' and children's attitudes toward active travel to school with positive feedback.
Sahlqvist, Veitch [50]	Australia	To study the impact of the campaign to promote active travel among children.	Primary school students	1 month	- Educational program: safety and traffic regulation. - Activities: walking campaign, rally games.	The campaign participants resulted in a slightly increased use of active travel mode in the short-term, average 1.4 trips per week compared to nonparticipants children.
Savolainen, Rutberg [51]	Sweden	Examine the experience and travel behavior change in participants in the AST intervention.	Primary school students	1 year	- Educational program: introduce the active travel mode, integrated into the lessons in each course. - Activities: games were conducted to encourage active transport (e.g., bingo games)	The results found that the combination of games and education, called gamification, led to the changeability of travel behavior among children and increased use of active transport.

### 4.1 Intervention description

Each study included interventions or activities tailored to the study area's specific characteristics or environments. Most education on the meaning and benefits of active travel was followed by practical activities to encourage active transportation in studies promoting active travel to school. Some studies employed the same intervention concept or campaign name, but the specific activities implemented in each area varied. For example, two studies utilized the STP campaign, a widely used and well-known campaign in Canada. However, the interventions implemented in these studies varied in focus. Buttazzoni, Clark [19] conducted their intervention in Canada, focusing mainly on education as the main activity, with other practical activities as a minor component. Meanwhile, Mammen, Stone [20] emphasized practical and real-world action, including realistic workshops and activities aimed at encouraging active travel, as well as enforcement strategies to change children's travel behavior. Furthermore, Christiansen, Toftager [47] reported investing in infrastructure to support and facilitate active transportation to and from schools, which was not commonly found in other interventions that focused on appropriating the built environment for active travel mode.

### 4.2 Effectiveness

After reviewing the 16 interventions presented in Table 1, we determined that most of them successfully promoted and encouraged active travel in children. Of the 16 studies, 14 demonstrated an increase in the ratio of nonmotorized travel mode after the intervention, indicating a significant change in travel behavior [19,20,30,[46], [47], [48], [49], [50], [51], [52]]. However, two studies reported intervention failure, showing no significant change in travel behavior between the baseline and follow-up or between the experiment and control groups [53,54]. The remaining study implemented a one-day intervention [45] that only showed transient effects and did not affect children's attitudes or behavior toward active travel in the long term beyond the intervention period.

## 5 Discussion

The results obtained in this review have substantial relevance to investigating interventions designed to promote active travel modes among children and adolescents. The review includes a diverse range of campaigns or interventions examined within the context of 16 reviewed articles that have been comprehensively interpreted in the overarching results section. Furthermore, the findings of this review show interesting outcomes across the reviewed articles. This section discusses each attribute that may influence the effectiveness of the promoted active travel mode intervention and the presentation of corresponding propositions for each identified issue.

### 5.1 Target population

Promoting active travel mode among children and adolescents can be challenging but worthwhile. Encouraging active travel at an early age can help children and adolescents develop independence and self-confidence by navigating their own transportation. In addition, it is important to provide them with the correct knowledge on using active travel modes safely. Children and adolescents are still physically and mentally developing, and promoting active travel modes can help establish early healthy habits [58]. Although adults can change their behavior slightly, they are often accustomed to the travel mode they have used for most of their lives [31]. Therefore, providing interventions to children and adolescents can lead to long-term changes in their travel behavior [30] Several studies have shown that changing attitudes and behaviors in adults is more difficult than in children or adolescents [59,60]. Consistent with the reviewed studies that used homogenous samples of elementary, primary, or secondary school students, this review has demonstrated the significant impact of providing interventions to children and adolescents on changing their travel behavior and attitudes toward active travel modes [30,46,48,52,55,56].

Proposition 1 *Promoting active travel modes among children and adolescents through interventions fosters independence*, *self-confidence*, *and healthy habits has a sustained impact on travel behavior*.

### 5.2 Implementation period

Several relevant factors must be considered to achieve a significant outcome or behavior change in children and adolescents receiving the intervention. Timing and frequency are essential factors that allow us to determine the success of the intervention [61]. As noted by Metcalf, Henley [62], the intervention period also affects the effectiveness of the provided intervention. Reviews indicate that the duration of the interventions implemented significantly impacts their results. For instance, short intervention periods, such as one-day or one-week programs, are typically insufficient to change attitudes and travel behavior in children and adolescents [45,54]. Conversely, longer and more consistent interventions have resulted in significant changes in travel behavior and increased use of active travel modes [20,30,41,[46], [47], [48], [49], [50], [51], [52],[55], [56], [57]]. Several studies have shown that repeating an activity for a sufficient period (e.g., a month, a trimester, half a year, etc.) will likely form a habit [[63], [64], [65], [66]].To maximize intervention effectiveness and ease of implementation, one should conduct the intervention twice a year, with each intervention cycle lasting three months, corresponding to the average length of a school semester. Such an approach would benefit the targeted population of children, adolescents, and educators, who are typically teachers.

Proposition 2 *Ensuring regular implementation over a sufficient period*, *typically twice a year for three months each time*, *increases the possibility of achieving the desired empirical outcome of an intervention*.

### 5.3 Intervention strategy

Accordingly, Buttazzoni, Clark [19] and Mammen, Stone [20] have demonstrated the significant effectiveness of the STP intervention in promoting active travel mode among children. However, one of the studies focused solely on education rather than practicality and discovered that the intervention had only a temporary impact [19]. A proper educational program is required to promote active travel mode in short-distance journeys to change the negative attitude toward it. Subsequently, it is critical to provide game-based activities that encourage and ensure confidence in active travel mode. Therefore, effective interventions or campaigns to promote active travel in children and adolescents should focus on both education and activities that motivate and encourage them to change their travel behavior, including their attitude toward nonmotorized modes. The essential contents in the educational intervention should make the participants realize the importance of using active travel modes in their short-distance journeys. This involves providing definitions and emphasizing the significance of active travel modes, including the benefits toward travelers, societies, and communities. Furthermore, the activities provided, such as walking/cycling rally games, milestone challenges, and others, must be engaging and entertaining enough for the children to participate without feeling bored or pressured [67]. Successful interventions often incorporate games or gamification concepts, awarding participants who achieve goals to stimulate, familiarize, and be encouraged to active travel mode, ultimately leading to a sustainable change in behavior [57,68,69]. Furthermore, the development of the intervention should consider safety issues by imparting basic safety knowledge for walking or cycling, including understanding traffic regulations. Some areas may have different environmental characteristics, such as bicycle lanes. Addressing safety content will inform travelers about traffic regulations in their surroundings. For example, in areas without designated bicycle lanes, they will know where they can safely bike or cycle, on pedestrian walkways or the shoulder of the road. Integrating safety content into the intervention can help reduce the risk of accidents during the trip.

Proposition 3 *Integrating appropriate education and gamification concepts into the intervention can improve its effectiveness and attractiveness*.

### 5.4 Intervention content versus outcomes

Several campaigns and interventions have been developed to promote active travel modes. The STP, which has been widely implemented in North America, is one of the most popular intervention programs, especially in Canada. Although the works of Mammen, Stone [20], Buttazzoni, Coen [41] used the same intervention program title, STP, to promote active travel mode among children, a closer examination of their content reveals significant differences. In particular, Buttazzoni, Coen [41] implemented a toolkit and games-based activities to motivate children to switch from other modes of transportation to active travel. Their study results showed that the intervention effectively achieved its objective. Meanwhile, Mammen, Stone [20] also designed a more practical intervention under STP that included education and game-based activities, workshops, and enforcement strategies. The results of their study showed that the intervention led to a higher proportion of change in travel behavior, which tended to be more lasting. Evidently, the difference of intervention content, educational program, and activities which even under the same title may provide significant different outcomes or effects (e.g., temporary effect and long-term effect). Additionally, the differences in the design and implementation of the interventions may arise from the characteristics of the participants and the built environment in the surrounding area. Therefore, the success of the intervention can be improved by taking into account context-specific factors and designing a tailored intervention that addresses the unique needs of the target population.

Proposition 4 *The difference in the content of the intervention can result in different outcomes*. *Practical activities might lead to a long-term effect of the intervention*.

### 5.5 The context of Thailand

**Fig. 2:**
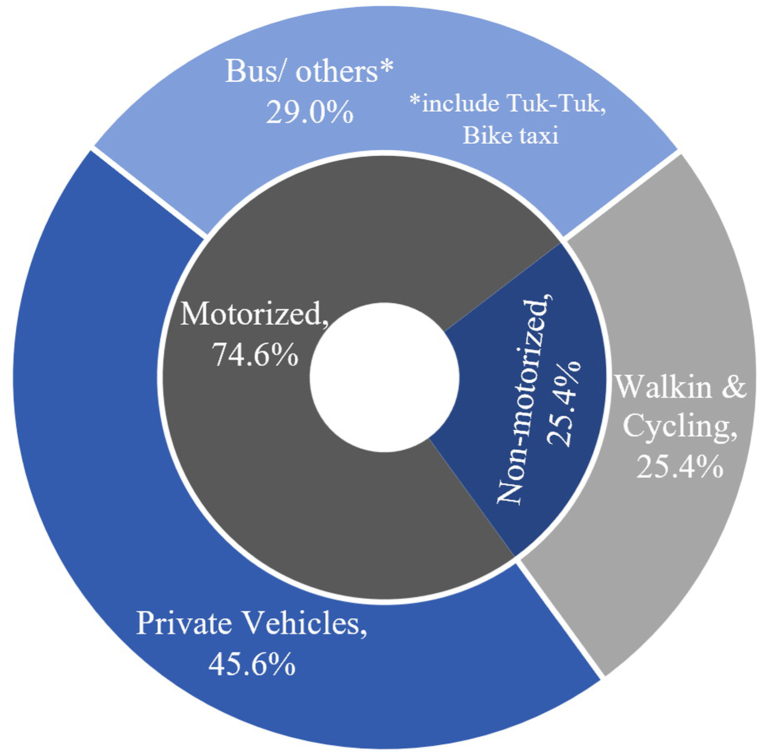
The modal share among adolescents aged between 13 and 18 years old in Thailand.

This type of study is typically conducted across many continents, except for Asia. There has been a dearth of appropriate campaigns or interventions to promote nonmotorized modes in Asia, particularly in Thailand, a developing Southeast Asian country. For many years, Thailand has had a problem with motorcycle riding behavior among adolescents, such as riding without a license and engaging in chaotic riding behavior. Furthermore, owning and riding a motorcycle is one of the most popular values among Thai adolescents [70]. As shown in Fig. 2, the modal share for short-distance journeys (1.5 km) among adolescents aged 13 to 18 is consistently dominated by private vehicles, including private cars and motorcycles. Riding a motorcycle has become a trend or a status symbol among high school students, whereas taking public transportation or walking/cycling to school is associated with a low socioeconomic status. On the contrary, using a private vehicle or motorcycle is considered convenient and indicative of a high socioeconomic standing. For example, individuals who possess or own their own vehicles will be more acceptable in society, while pedestrians would be treated vice versa. Therefore, providing interventions to encourage adolescents to adopt active travel modes for short-distance travels may lead to a significant shift in the modal share by a considerable proportion.

Proposition 5 *Values associated with private vehicle ownership*, *socioeconomic status*, *and negative attitudes toward active travel modes among adolescents can substantially impact the nonmotorized modal share of travel*.

## 6 Conclusions

This study presents interventions to encourage children and adolescents to use active travel mode. The interventions identified in this review are primarily composed of education and activities that encourage the use of active travel modes. Considering the Thai context, this study found that the intervention period should not be too short, twice a year, with a duration of 3 months for each intervention cycle, aligning with the average length of a school semester. The intervention should be provided regularly for long-term sustainable success in transitioning to an active travel mode.

In terms of activities designed to encourage active travel for short-distance travels, game-based concepts such as Travel Bingo can be integrated, where participants cross out the objects they encounter while walking or cycling to school. Small rewards or prizes should be given to those who win or meet the goal to incentivize and encourage the transition to active modes of transportation. Furthermore, simulator games such as Walking Rally or Cycling Rally can be used to simulate the actual circumstances that arise when children use active transportation, preparing them to deal with any challenges that may arise along the way. Launching such activities aims to introduce children and adolescents to active transportation, eventually encouraging it to become a habit.

However, changing attitudes and mindsets requires education and promoting active travel modes. Educational interventions must include information on the advantages and practicalities of non-motorized travel modes. Furthermore, concerns about safety and traffic should be addressed. These topics could be incorporated into current school curriculum lessons. As a result, educational interventions must focus on children and adolescents to overcome their negative attitudes and mindsets toward active transportation modes while eliminating any inequality among users of such modes.

Therefore, a practical approach to learning by doing might be the optimal alternative. The findings of this study can serve as a guide for designing effective campaigns or interventions aimed at promoting active travel among children and adolescents. The developers may take these findings to integrate and adapt them into their campaigns or intervention strategies, which will lead to a change in the travel behavior of children or adolescents. Furthermore, to ensure that interventions that promote active travel mode are sustainable and permanent, the support of the government and the related public sector is essential. The successful implementation of the intervention necessitates the provision of policy and infrastructure support. Specifically, within the context of Thailand, it is imperative to enhance pedestrian walkways and associated facilities that cater to active travel, such as the improvement of bike lanes. Ensuring equitable treatment of pedestrians toward other modes of transportation requires the rigorous enforcement of laws pertaining to pedestrians and cyclists. Hence, to delve deeper and potentially enhance the intervention's effectiveness, more attention is needed on other aspects that influence travelers to choose active travel modes, such as the built environment and facilities surrounding schools and communities. Such research is crucial, as it is a key factor influencing the adoption of this mode of transportation. This approach will ensure the sustainability of the campaign in the long run.

## Data availability statement

No data was used for the research described in this article.

## CRediT authorship contribution statement

**Kattreeya Chanpariyavatevong:** Writing – review & editing, Writing – original draft, Validation, Methodology, Formal analysis, Conceptualization. **Thanapong Champahom:** Validation. **Chamroeun Se:** Validation. **Sajjakaj Jomnonkwao:** Writing – review & editing, Methodology. **Vatanavongs Ratanavaraha:** Supervision, Project administration.

## Declaration of competing interest

The authors declare that they have no known competing financial interests or personal relationships that could have appeared to influence the work reported in this paper.
